# Event-related potentials reflect prediction errors and pop-out during comprehension of degraded speech

**DOI:** 10.1093/nc/niaa022

**Published:** 2020-10-25

**Authors:** Leah Banellis, Rodika Sokoliuk, Conor J Wild, Howard Bowman, Damian Cruse

**Affiliations:** School of Psychology and Centre for Human Brain Health, University of Birmingham, Edgbaston B15 2TT, UK; School of Psychology and Centre for Human Brain Health, University of Birmingham, Edgbaston B15 2TT, UK; Brain and Mind Institute, University of Western Ontario, London, ON N6A 3K7, Canada; School of Psychology and Centre for Human Brain Health, University of Birmingham, Edgbaston B15 2TT, UK; School of Computing, University of Kent, Canterbury, Kent CT2 7NF, UK; School of Psychology and Centre for Human Brain Health, University of Birmingham, Edgbaston B15 2TT, UK

**Keywords:** conscious pop-out, prediction error minimization, global neuronal workspace breakthrough

## Abstract

Comprehension of degraded speech requires higher-order expectations informed by prior knowledge. Accurate top-down expectations of incoming degraded speech cause a subjective semantic ‘pop-out’ or conscious breakthrough experience. Indeed, the same stimulus can be perceived as meaningless when no expectations are made in advance. We investigated the event-related potential (ERP) correlates of these top-down expectations, their error signals and the subjective pop-out experience in healthy participants. We manipulated expectations in a word-pair priming degraded (noise-vocoded) speech task and investigated the role of top-down expectation with a between-groups attention manipulation. Consistent with the role of expectations in comprehension, repetition priming significantly enhanced perceptual intelligibility of the noise-vocoded degraded targets for attentive participants. An early ERP was larger for mismatched (i.e. unexpected) targets than matched targets, indicative of an initial error signal not reliant on top-down expectations. Subsequently, a P3a-like ERP was larger to matched targets than mismatched targets only for attending participants—i.e. a pop-out effect—while a later ERP was larger for mismatched targets and did not significantly interact with attention. Rather than relying on complex *post hoc* interactions between prediction error and precision to explain this apredictive pattern, we consider our data to be consistent with prediction error minimization accounts for early stages of processing followed by Global Neuronal Workspace-like breakthrough and processing in service of task goals.

## Introduction

Prediction error minimization accounts of perception propose that the brain seeks to minimize the mismatch between incoming sensory information and top-down expectations (Rao and Ballard 1999; Friston 2010). To successfully comprehend speech, a prediction error minimization account argues that the listener must generate a set of expectations at multiple levels of representation to attempt to most accurately explain the auditory input (Paczynski and Kuperberg 2012). Consistent with the role of expectations in speech comprehension, the amplitude of the N400 event-related potential (ERP) in response to the final word of a sentence increases with how unexpected that word is, given the context of the sentence (Kutas *et al.* 1984; Kutas and Federmeier 2011). The N400 can, therefore, be characterized as an index of the amount of mismatch between a semantic prediction and the incoming stimulus—i.e. a semantic prediction error (Paczynski and Kuperberg 2012; Bornkessel-Schlesewsky and Schlesewsky 2019). Indeed, prediction error minimization accounts of global brain function, such as free energy (Friston 2010), propose that all evoked activity in the brain reflects this mismatch of prediction and stimulus, i.e. the prediction error (Rao and Ballard 1999; Clark 2013).

However, not all ERPs can be characterized parsimoniously within a narrow prediction error framework. For example, highly predictable events in rapid serial visual presentation (RSVP) that are associated with a subjective experience of conscious ‘breakthrough’ or ‘pop-out’ also elicit large ERPs from ∼300 ms post-stimulus (i.e. the P300; Donchin and Coles 1988), while unpredictable events in the same stream of stimuli will elicit almost no evoked response (Bowman *et al.* 2013; Rohaut *et al.* 2015)—the opposite of what would be predicted if ERPs indexed prediction error only. To account for these apparently apredictive effects, prediction error minimization accounts propose that attention increases the precision of predictions, and that prediction error is subsequently weighted by this precision (Kok *et al.* 2012). As a result, a range of ERP magnitudes, including late apredictive components such as the P300 in RSVP, can be explained as contributions from independently varying precision and prediction error (see also Heilbron and Chait 2018).

The Global Neuronal Workspace is an alternative theory of neural processing that proposes that such apredictive evoked positivities with onsets ∼300 ms post-stimulus reflect the ignition of a stimulus representation into a frontoparietal network for conscious access—whether that stimulus was or was not expected—while earlier ERPs index preconscious processes, including prediction errors (Dehaene *et al.* 1998; Sergent *et al.* 2005). Applying this model to speech comprehension, Rohaut *et al.* (2015) proposed a two-stage ERP profile, with an initial unconscious semantic prediction error in response to each word (the N400, typically onsetting around 200 ms post-stimulus) and a late positive complex (LPC; in this case onsetting around 600 ms post-stimulus) reflecting the ignition of meaning into conscious access. In support of this proposal, the N400 ERP has been observed in states of relative unawareness such as sleep, coma and vegetative state (or unresponsive wakefulness syndrome) (Kotchoubey *et al.* 2005; Ibáñez *et al.* 2006; Rämä *et al.* 2010; Beukema *et al.* 2016) while the LPC has only been reported in conscious individuals, or in those who were conscious of and subsequently could report target words (Sergent *et al.* 2005; van Gaal *et al.* 2014; Rohaut *et al.* 2015).

We sought to test the proposal that early ERPs (<300 ms post-stimulus) reflect preconscious prediction error processes and later ERPs (>300 ms post-stimulus) reflect conscious access by investigating the comprehension of speech that has been degraded by noise-vocoding (Shannon *et al.* 1995). Consistent with the role of expectations in speech comprehension, a noise-vocoded speech stimulus that is entirely unintelligible to a naive listener can be rendered intelligible through priming—e.g. by presenting a non-degraded version of the stimulus (i.e. a *matched* prime) immediately prior to the degraded stimulus (i.e. the target). When successfully primed in such a word-pair listening task, listeners experience a ‘pop-out’ of the meaning of the degraded speech—i.e. subjective conscious access (Davis *et al.* 2005)—while an unrelated (or, *mismatched*) prime will not facilitate comprehension of the subsequent target.

Evidence suggests that successful comprehension of noise-vocoded speech requires attentional effort (Hervais-Adelman *et al.* 2012) and top-down expectations from frontal lobes (Sohoglu *et al.* 2012; Wild *et al.* 2012b). Therefore, we predict that if successful comprehension of degraded speech requires effortful generation of top-down expectations, distracted participants will be unable to use a prime word to generate an expectation of the identity of an upcoming target, and will, therefore, neither exhibit a differential word identity prediction error signal nor any subsequent apredictive evoked response to the target. Conversely, we expect that attentive participants will use the prime to generate top-down expectations of the identity of the degraded stimulus, and will therefore more readily comprehend the target. Consequently, and consistent with a two-stage Global Neuronal Workspace account (Rohaut *et al.* 2015), we expect attentive participants’ ERPs to exhibit an initial prediction error signal [i.e. larger evoked response to mismatched targets; cf. Sohoglu *et al.* (2012)] followed by an apredictive ‘pop-out’ effect in which the ERP to the correctly expected and comprehended targets is larger than that to the unexpected and predominantly unintelligible targets.

## Materials and Methods

### Participants

We recruited participants from the University of Birmingham via advertisement on posters or the online SONA Research Participation Scheme until we had achieved our desired sample size of 48 participants with usable data (24 per group; median age = 20 years, range = 18–33 years). While we did not conduct a formal *a priori* power analysis, we chose a sample of 24 per group as this is approximately double the size of samples in similar previous studies (e.g. Sohoglu *et al.* 2012) and allowed us to fully counterbalance stimuli lists across participants (see Procedure section below). Our inclusion criteria were right-handed (from self-report), 18–35 years old, monolingual speakers of British English, with no self-reported epilepsy, dyslexia or uncorrected hearing impairment. We compensated participants with course credit or £10/h of their time. The STEM Research Ethics Board of the University of Birmingham granted ethical approval for this study and written informed consent was completed by all participants. To achieve our final sample, we recruited 77 participants but rejected data from 29 participants due to an error of randomization in the experimental code.

### Stimuli

A male first-language British English speaker recorded 288 monosyllabic English nouns taken from previous priming studies in our lab (see https://osf.io/m9ud5/ for the full stimuli list; mean length = 440 ms, range = 264–657ms, sampling rate = 44 100 Hz). First, we randomly assigned the stimuli to one of four equal-sized lists (72 words per group) and manually swapped words across lists until the lists were matched on imageability, frequency (BNC), length in phonemes and length in letters. Frequentist tests (ANOVAs) indicated no evidence that the four lists differed in word frequency [*F*(3, 284) = 0.233, *P *=* *0.873], imageability [*F*(3, 231) = 0.779, *P *=* *0.507], length in phonemes [*F*(3, 284) = 0.217, *P *=* *0.885] or length in letters [*F*(3, 284) = <0.001, *P *=* *1; see Supplementary data], and Bayesian equivalent tests [conducted with JASP Team (n.d.) and Morey and Rouder (n.d.)] revealed strong evidence in favour of the null hypothesis for all variables (all BF10 = <0.05; see Supplementary data). From these four matched lists, we created counterbalanced conditions across participants (see Procedure section).

We manipulated the intelligibility of targets through noise-vocoding—originally a form of auditory distortion used to simulate the experience of hearing by the means of a cochlear implant (Shannon *et al.* 1995) (for scripts see https://github.com/conorwild/matlab-audio-scripts/). Noise-vocoding retains the coarse temporal structure of the speech but reduces spectral clarity and fine temporal detail. The amplitude envelope from (approximately) logarithmically spaced frequency bands is extracted and applied to bandpass-filtered noise of the same frequency band. Finally, the bands of envelope-modulated noise are recombined to create the final noise-vocoded stimulus (Davis *et al.* 2005). Using this method, we created six-band noise-vocoded versions of each stimulus to be used as the targets, and subsequently normalized each stimulus to its root mean square (RMS; Wild *et al.* 2012a,b; see Supplementary data for auditory examples of word stimuli).

### Procedure

We randomly assigned each participant to be in the attentive or distracted group. All participants simultaneously heard the same auditory stimuli and viewed the same visual stimuli, with some small differences in inter-stimulus intervals between groups to allow for behavioural responses (detailed below). Those in the attentive group paid attention to, and responded to, the auditory stimuli, while those in the distracted group paid attention to, and responded to, the visual stimuli.

**Figure 1.**
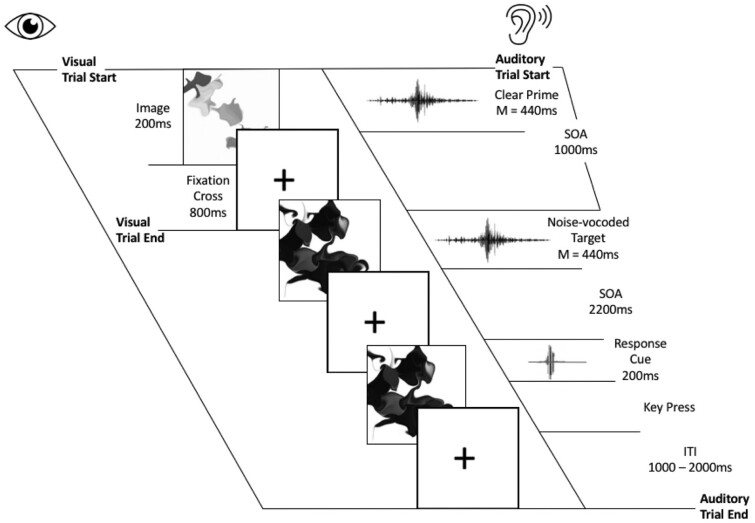
Schematic of event timing for participants in the attentive group who were instructed to attend and respond to the auditory stimuli while ignoring the visual stimuli. Participants in the distracted group, conversely, were instructed to attend and respond to the visual stimuli while ignoring the auditory stimuli. Timings for the distracted group were identical to those shown in the figure, with the exception that there was no auditory response cue or wait period for a key press

In the attentive group, each auditory trial began with the auditory presentation of the prime followed by the target with a stimulus onset asynchrony of 1 s (see Fig. 1). After 2.2 s, participants were cued by a tone (500 Hz, 200 ms duration) to rate the ‘noisiness’ of the target on a scale of 1 (low) to 5 (high) via keyboard press. Following each rating, the next trial began after an inter-trial interval of between 1 and 2 s, selected randomly from a uniform distribution on every trial.

In the distracted group, participants listened to the same auditory stimuli but did not complete the noisiness judgement task; instead, they made responses to the visual stimuli (see below). Therefore, the timing of the auditory stimuli in the distracted group was identical to the attentive group, with the exception that the time between the onset of each auditory target and the onset of the next auditory trial did not include a waiting period for a behavioural response. Rather, the inter-trial interval was between 1 and 2 s, selected randomly from a uniform distribution on every trial.

While listening to the auditory stimuli, both attentive and distracted groups of participants watched a sequence of rapidly changing visual stimuli. However, only those in the distracted group were instructed to complete a task on the basis of the visual stimuli and to ignore the auditory stimuli. The distraction task was a 1-back visual monitoring task, in which the sequence of visual stimuli was comprised of a series of images of ambiguous black shapes presented on a white background. Each ambiguous image was shown for 200 ms with an 800 ms fixation period between each image. For each participant, the order of images was randomized and the task for those in the distracted group was to press a key every time a repetition occurred (i.e. a 1-back task; 20% of trials). We subsequently calculated task accuracy to ensure that participants in the distracted group were distracted from the auditory stimuli by attending to the visual 1-back task. Participants in the attentive group watched the same visual stimuli, but were instructed to ignore them and to attend to the auditory stimuli only.

Upon completion of the above task, all participants completed a surprise recognition memory test—i.e. they were presented with a subset of previously heard (old) words interspersed with (new) words that they had not heard previously and were required to judge each word’s old/new status. The memory test stimuli were formed of all 144 words from the mismatched condition of the word-pair priming task (i.e. 72 primes and 72 targets), as well as 72 new memory test items. We did not include the matched targets in the memory test as they had been presented twice (as a clear prime and as a degraded target) and therefore cannot be compared to the unrelated targets or primes which had only been presented once. In the memory test, each word was presented visually for 300 ms, with a fixation point present for 2–3 s (selected randomly from a uniform distribution on each trial) between each word. We randomized the order of words for each participant. Participants made an old/new discrimination for each word on a 6-point remember-know scale, made up of the following responses; ‘definitely new, probably new, not sure, probably old, definitely old and remember’ (Ritchey *et al.* 2015). We reversed the scale and inverted the responses for half of the participants to control for potential effects of response hand and thus remove any motor preparation differences. Note that electroencephalography (EEG) data acquired during the memory task are not analysed here and are beyond the scope of this article. Nevertheless, all data are available in the online repository for analysis by the community (https://osf.io/m9ud5/).

During the experiment, each participant heard all four word lists (see Stimuli section), with each list comprising either the matched words, mismatched primes, mismatched targets or the new memory test items. For each of the 12 possible combinations of word lists for mismatched primes and mismatched targets, we manually ensured that there was no phonological, semantic or associative overlap between the target and the prime. In total, there were 24 possible sets of stimuli to achieve full counterbalancing of lists. Therefore, across all participants, each word was heard an equal number of times in every possible condition.

### EEG pre-processing

We recorded EEG with a 128-channel Biosemi ActiveTwo system at a sample rate of 256 Hz, with two additional electrodes recording from the mastoid processes. Offline, we digitally filtered the EEG signal between 0.5 and 40 Hz, segmented the data into epochs from 500 ms before the onset of the prime until 1000 ms after the onset of the target, re-referenced the data to the average of the mastoids, and baseline corrected to the 200-ms pre-prime period. Unless otherwise stated, all offline pre-processing was performed with a combination of the Matlab toolbox EEGLAB (version 14.0.0b; Delorme and Makeig 2004) and custom scripts. Note that all scripts are available online at https://osf.io/m9ud5/

Artefact rejection proceeded in the following steps. First, we used an automated procedure, based on FASTER (Nolan *et al.* 2010), to identify and remove bad channels. Specifically, bad channels were those with absolute z-scores of >2.5 on any of the following measures: variance of voltage, mean correlations with other channels and Hurst exponent. Across participants, a median of seven channels was discarded (range: 2–14). Second, we used an automated procedure, also based on FASTER (Nolan *et al.* 2010), to identify and remove trials with non-stationary artefacts. Specifically, a trial was bad if its absolute z-score was >2.5 on any of the following measures: mean range of voltages across channels, mean variance of voltages across channels and the deviation of the trial average voltage from the average voltage across all channels. Third, we conducted independent component analysis of the remaining data (EEGLAB’s *runica* algorithm) and used the toolbox ADJUST (Mognon *et al.* 2011) to automatically identify and remove components with the expected spatial and temporal features of blinks, eye-movements and generic discontinuities. Next, we interpolated any previously removed channels back into the data. Finally, trials with artefacts that had not been effectively cleaned by the above procedure were identified with visual inspection and discarded. After these pre-processing steps, a median of 65.5 trials contributed to the match condition (range: 39–71) and a median of 66 trials contributed to the mismatch condition (range: 37–71). Prior to analysis, all data were re-referenced to the average of all channels.

For our subsequent memory ERP contrasts, we only included data from the attentive group as recognition memory was not significantly greater than chance for the distracted group. We also excluded those participants who contributed fewer than 12 trials to either of the two categories [i.e. targets that were subsequently remembered (hits) versus targets that were subsequently forgotten (misses)] and those whose recognition memory was not greater than zero, resulting in a subgroup of 13 participants (hits: median 21, range 13–36; misses: median 26, range 12–49).

### EEG/MRI co-registration

The electrode locations of each participant were recorded relative to the surface of the head with a Polhemus Fastrak device using the Brainstorm Digitize application (Brainstorm v. 3.4; Tadel *et al.* 2011) running in Matlab (Mathworks). Furthermore, on a separate day, we acquired a T1-weighted anatomical scan of the head (nose included) of each participant with a 1 mm resolution using a 3T Philips Achieva MRI scanner (32 channel head coil). This T1-weighted anatomical scan was then co-registered with the digitized electrode locations using Fieldtrip (Oostenveld *et al.* 2011).

### Sensor analyses: ERPs

Prior to analysis, we calculated participant-wise average ERPs for each condition separately using the robust averaging method of SPM12 (default params) that iteratively down-weights outlier voltages across trials. As recommended in the SPM12 documentation, the subsequent average ERPs were then low-pass filtered at 20 Hz (i.e. a second low-pass filter after the 40 Hz pre-processing filter), and baseline corrected to the 200 ms prior to the onset of the target.

**Figure 2.**
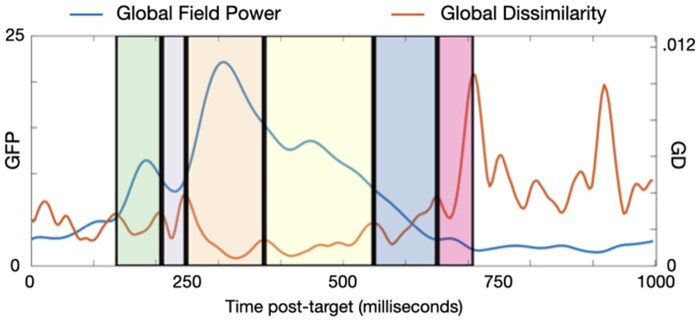
GFP and Global Dissimilarity of the average of all trials (i.e. an orthogonal contrast) in the post-target window, with the time windows of interest highlighted. Peaks of GFP separated by peaks of Global Dissimilarity are indicative of distinct evoked components

Time window selection for the ERP analyses proceeded in two stages, and in a similar way to (Sohoglu *et al.* 2012). First, we calculated the global field power (GFP) (Skrandies 1990) of the grand average of all trials (i.e. both conditions together) to identify time windows of interest. GFP is the root mean square of average-referenced voltages and is a principled means of identifying component peak latencies from an orthogonal contrast (Skrandies 1990). We then identified a time window around each peak by inspecting the global dissimilarity (Skrandies 1990)—the mean of the root mean square of voltage differences between consecutive time points, after the data have been scaled by the GFP. Deflections in the time course of global dissimilarity therefore suggest boundaries between scalp topographies. On this basis, we selected the following ERP topographies: 137–207, 211–246, 250–371, 375–547, 551–648 and 652–707 ms (Fig. 2).

To minimize the number of comparisons in post-target time windows, we only investigated the main effects of target type (i.e. matched versus mismatched, averaged across attention group) and attention (i.e. attention versus distraction, averaged across target type) when an interaction contrast produced no significant clusters (i.e. the difference between matched targets and mismatched targets between attention groups). Where a significant interaction cluster was observed, we tested for simple effects with paired samples *t*-tests of data averaged within the electrodes that contributed to the interaction cluster. Furthermore, within each significant interaction and main effect cluster, we investigated subsequent memory effects (hits versus misses) with paired samples *t*-tests of data averaged within the electrodes that contribute to each cluster.

ERPs (or difference ERPs between two within-subject conditions) within each time window of interest were compared with the cluster mass method of the open-source Matlab toolbox FieldTrip (version 20160619, Oostenveld *et al.* 2011). First, for each participant × condition, we averaged the voltages at each electrode within the time window of interest. Next, a two-tailed *t*-test (dependent samples for interaction and main effect of target type; independent samples for main effect of attention) between conditions was conducted at each electrode. Spatially adjacent *t*-values with *P*-values passing the threshold (alpha = 0.05) were then clustered based on their spatial proximity. Clusters were required to involve at least four neighbouring electrodes, with an electrode’s neighbourhood defined as all electrodes within ∼4 cm on a template head (median number of neighbours: 11; range: 2–16). A second non-parametric step corrects for multiple comparisons by conducting 1000 Monte Carlo randomizations of the above method (shuffling condition labels) to estimate the probability of the observed cluster under the null hypothesis (Maris and Oostenveld 2007). We applied a cluster alpha threshold of 0.025 as we were testing for both positive and negative effects.

### Sensor analyses: Bayesian tests

When the above sensor analyses failed to find support for an interaction between target type and attention (i.e. the difference of differences) but did find evidence of a main effect, we used Bayesian equivalent *t*-tests to test the sensor data for evidence in support of the null hypothesis. Specifically, at each electrode, we calculated a Jeffrey–Zellner–Siow Bayes factor (JZS-BF), implemented with an open-access script (https://github.com/anne-urai/Tools/tree/master/stats/BayesFactors). A JZS-BF between 1 and 3 is considered to be weak/anecdotal evidence in support of the hypothesis being tested; from 3 to 10 is substantial evidence; and 10 to 100 is strong evidence (Jeffreys 1961). Note that, as the Bayes Factor is the ratio of evidence for two hypotheses, the same category descriptions hold for the inverse (i.e. 1/3, 1/10, 1/100). While this approach does not take into account spatial clustering, as in the sensor analyses above, it does allow us to qualitatively inspect the spatial distribution of evidence in support of the null hypothesis across the head.

### Source estimation

We performed source estimation using EEG data and individual electrode locations from 48 participants. The analyses were completed using subject-specific T1-weighted anatomical magnetic resonance imaging (MRI) scans for 39 participants and template T1-weighted MRI images (provided by the Matlab toolbox FieldTrip) for the remaining nine participants due to issues with T1 data collection and image quality.

From the subject-specific T1-weighted anatomical scans, individual boundary element head models (four layers) were constructed using the ‘dipoli’ method of the Matlab toolbox FieldTrip (Oostenveld *et al.* 2011). Individual electrode locations were aligned to the surface of the scalp layer extracted from the segmented T1-weighted anatomical scans using fiducial points and head shape as reference points. The alignment of electrodes and scalp surface was further visually inspected to detect potential deviations and, where necessary, small manual corrections were applied.

As we required single-trial data to estimate the sources of the ERP effects, we used the pre-processed sensor-level data prior to the robust averaging step described above. We defined trials as time windows from −500 to 1900 ms relative to the prime, and baseline corrected the EEG data using the time window (−200 to 0 ms) relative to target presentation (i.e. the same time window as for the sensor analyses). Before the direct statistical comparison, we balanced the number of trials between conditions by randomly removing trials from the condition (discarded trials: median = 2, range = 0–13) with more data until both datasets had the same number of trials (median = 130, range = 74–136).

Our source estimation followed the analysis approach described in Popov *et al.* (2018). Therefore, the data was first filtered between 1 and 40 Hz, using a first filter as implemented in the *ft_preprocessing* function of Fieldtrip (using default parameters). Additionally, to mitigate the confounding influence of correlated activity in the auditory cortices (i.e. from binaural stimulation) on linearly constrained minimum variance (LCMV) beamformer source analysis, we calculated the surface Laplacian of the data and leadfields as in Murzin *et al.* (2013). Thus, the scalp current density of the data was calculated and the covariance matrix was estimated using a time window from −500 to 1900 ms. A common spatial filter (including trials of both conditions) was computed using an LCMV beamformer (inputting the surface Laplacian transformed leadfield) (Van Drongelen *et al.* 1996; Van Veen *et al.* 1997; Robinson and Vrba 1999). Specific beamformer parameters were chosen based on the approach used by Popov *et al.* (2018) including a fixed dipole orientation, a weighted normalization (to reduce the centre of head bias), as well as a regularization parameter of 5% to increase the signal-to-noise ratio. This common spatial filter was then used for source estimation. The dipole moments of both conditions were extracted in the post-stimulus time windows that showed significant clusters at the sensor level (time window 1: 137–207 ms; time window 2: 211–246 ms; time window 3: 250–371 ms; time window 4: 551–648 ms), and their absolute values were averaged over time points to obtain one average value per grid point (virtual electrode) and time window of interest. For clear visualization of the foci of our source estimates, we calculated *t*-tests at each virtual electrode and thresholded the subsequent *t*-images at *P* < 0.05 [see Supplementary data and Sokoliuk *et al.* (2019), for further validation of the method].

## Results

### Speech intelligibility

Attentive participants rated the mismatched targets as noisier (median = 4, range 1–5) than the matched targets (median = 3, range 1–4), despite the stimuli being physically distorted to the same level. This difference was significant in a Wilcoxon Signed-Rank Test (*W* = 232, *P* < 0.001).

### Recognition memory: discrimination (*d*ʹ)

To quantify participants’ ability to discriminate between old and new items in the memory test, we calculated their discrimination score (*d*ʹ). dʹ was calculated as the z-transformed proportion of hits (i.e. ‘old’ responses to old items) minus the z-transformed proportion of false alarms (i.e. ‘old’ responses to new items; Haatveit *et al.* 2010). The proportion of hits and false alarms was transformed using the inverse of the standard normal cumulative distribution. All ‘probably old’, ‘definitely old’ and ‘remember’ responses to old items were considered a hit, while the same responses to new items were considered false alarms.

A two-way mixed ANOVA with factors of word type (clear prime; degraded target; both only heard once by each participant) and attention (attentive; distracted) revealed significant main effects of word type [*F*(1, 46) = 30.243, *P* = ≤ 0.001, partial *n*^2^ = 0.397] and attention [*F*(1, 46) = 8.714, *P* = 0.005, partial *n*^2^ = 0.159], and a non-significant interaction [*F*(1, 46) = 0.528, *P* = 0.471, partial *n*^2^ = 0.011]. A Bayesian equivalent mixed ANOVA revealed considerable evidence for a model containing main effects of both word type and attention (BF = 69 083 relative to a null model), which itself was 2.687 times more likely given the data than a model containing both main effects and an interaction term. These results reflect the participants’ more accurate memory for clear primes than degraded targets and the higher memory accuracy in the attentive group than the distracted group (see Supplementary data for all inferential statistics).

One-sample *T*-tests determined that *d*ʹ for both clear primes and degraded targets were significantly different from zero (i.e. above chance) for attentive participants [clear: mean dʹ= 0.457, SD = 0.309, *t*(23) = 7.245, *P* ≤ 0.001; degraded: mean dʹ = 0.194, SD = 0.218, *t*(23) = 4.368, *P* ≤ 0.001], while memory for either item type was not significantly different from zero for distracted participants [clear: mean = 0.132, SD = 0.515, *t*(23) = 1.251, *P* = 0.223; degraded: mean = −0.070, SD = 0.393, *t*(23) = −0.869, *P* = 0.394], suggesting that the distraction task effectively suppressed processing of the auditory stimuli. Bayesian equivalent *T*-tests indicated considerable evidence for better than chance memory for attentive participants (clear: BF10 = 71 004; degraded: BF10 = 131), and anecdotal evidence for chance-level memory performance for distracted participants (clear: BF10 = 0.430; degraded: BF10 = 0.302).

### Recognition memory: Recollection and Familiarity

To estimate the level of processing that the clear and degraded words received, we calculated separate measures of Recollection (i.e. explicit contextualized memory of the event) and Familiarity (i.e. memory without context) from the recognition memory judgements for each participant (Atkinson and Juola 1974; Yonelinas *et al.* 1997). Specifically, Recollection scores were calculated by: (Rold – Rnew)/(1 – Rnew), with Rold reflecting the proportion of old items given a Remember response by the participant, and Rnew reflecting the proportion of new items given a Remember response. Familiarity was calculated by: [Fold/(1 − Rold)] − [Fnew/(1 − Fnew)], with Fold reflecting the proportion of old items given a ‘definitely old’ or ‘probably old’ response by the participant, and Fnew reflecting the same responses to new items (Ritchey *et al.* 2015).

Two two-way mixed ANOVAs with factors of word type (clear prime; degraded target; both only heard once by each participant) and attention (attentive; distracted) revealed significant main effects of word type on both Recollection and Familiarity estimates [*F*(1, 46) = 13.287, *P* ≤ 0.001, partial *n*^2^ = 0.224, and *F*(1, 46) = 14.533, *P* ≤ 0.001, partial *n*^2^ = 0.240, respectively], reflecting higher scores for clear primes than for degraded targets. No other main effect or interaction was significant (all *P*s > 0.127). Bayesian equivalent ANOVAs similarly concluded that there was strong evidence for models containing a main effect of word type for both Recollection (BF10 = 43.703, BFinclusion = 60.743) and Familiarity (BF10 = 64.090, BFinclusion = 42.913).

One-sample *t*-tests identified significantly different from zero measures of Recollection for clear primes [*t*(23) = 6.127, *P* ≤ 0.001, BF10 = 6538.041] and degraded targets [*t*(23) = 2.499, *P* = 0.020, BF10 = 2.720] in the attentive group, while Familiarity was not different from zero [clear: *t*(23) = −0.009, *P* = 0.993, BF10 = 0.215; degraded: *t*(23) = −1.899, *P* = 0.070, BF10 = 0.998]. In the distracted group, neither Recollection nor Familiarity were significantly different from zero for either word type [Recollection clear: *t*(23) = 0.245, *P* = 0.809, BF10 = 0.221; Recollection degraded: *t*(23) = −1.003, *P* = 0.326, BF10 = 0.337; Familiarity clear: *t*(23) = −1.452, *P* = 0.160, BF10 = 0.542; Familiarity degraded: *t*(23) = −2.042, *P* = 0.053, BF10 = 1.247].

### Event-related potentials

#### Interaction effects

**Figure 3.**
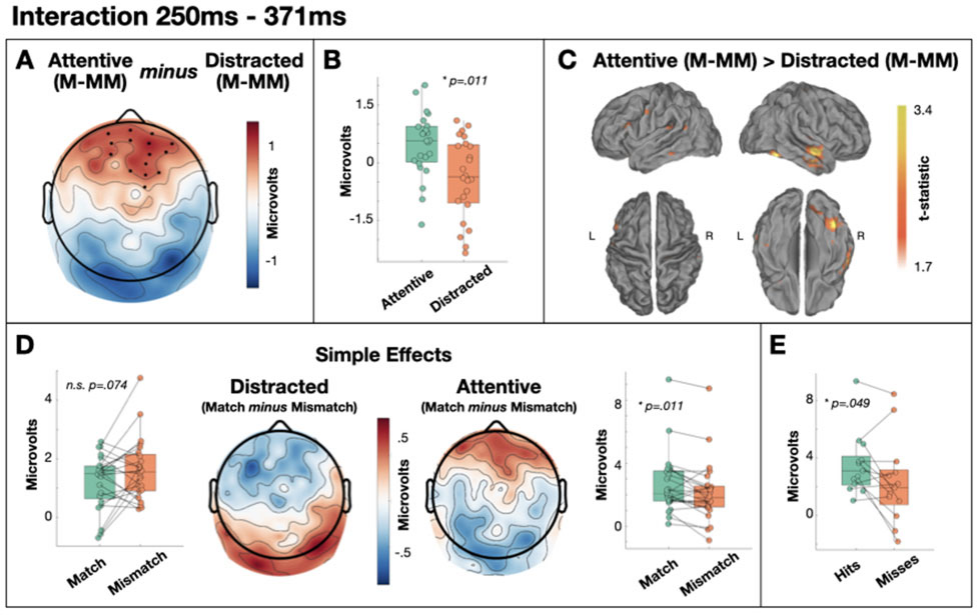
Interaction between target type and attention from 250 to 371 ms. (**A**) Scalp distribution of the significant difference in the effect of target across attention conditions. Electrodes contributing to the cluster are marked. M-MM, match minus mismatch. (**B**) Single-subject mean difference voltages (difference between match and mismatch) within the significant cluster. (**C**) Estimated sources of the attentive effect of target in right middle temporal gyrus and right fusiform gyrus (relative to the distracted effect of target). (**D**) Analysis of the simple effects showing qualitatively different topography across attention groups, and a significant effect of target type in the attentive group only. (**E**) Subsequent memory effect within the interaction cluster

We observed an interaction between target type and attention in the 250–371 ms time window post-target only (cluster *P* = 0.011) with estimated generators in right middle temporal gyrus and right fusiform gyrus (Fig. 3). Follow-up simple effects tests indicated greater positivity within this cluster for matched targets relative to mismatched targets during auditory attention only [*t*(23) = 2.755, *P* = 0.011, two-tailed, BF10 = 4.376; Fig. 3D]. While the mean voltages in this time window exhibited the opposite pattern in the distracted group—i.e. greater positivity to mismatched targets—this difference did not pass our significance threshold [*t*(23) = −1.869, *P* = 0.074, two-tailed; Fig. 3D] and a Bayesian equivalent analysis found only anecdotal evidence in favour of the null hypothesis in this contrast (BF10 = 0.955). A subsequent memory contrast within this cluster indicated greater positivity for mismatched targets that were subsequently remembered (hits) relative to mismatched targets that were subsequently forgotten (misses) in the attentive group, although this effect was only weakly significant in a *t*-test [*t*(12) = 2.185, *P* = 0.049, two-tailed; Fig. 3E] and a Bayesian equivalent indicated that the evidence was only anecdotal (BF10 = 1.628). No clusters were formed in any other time window for the interaction contrast. We therefore examined the main effects below.

#### Main effects

**Figure 4.**
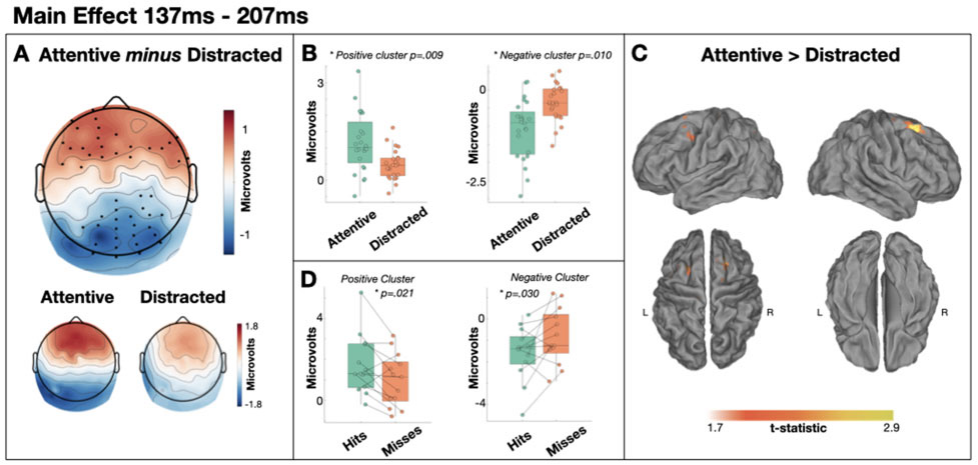
Main effect of attention from 137 to 207 ms. (**A**) Scalp distribution of the significant effect. Electrodes contributing to the clusters are marked. (**B**) Single-subject mean voltages within each significant cluster. (**C**) Estimated sources of the main effect within right superior frontal lobe. (**D**) Subsequent memory effects within each cluster in the attentive group

We observed a dipolar main effect of attention in the 137–207 ms time window, with greater frontal positivity (cluster *P* = 0.009) and greater posterior negativity (cluster *P* = 0.010) for attentive participants relative to distracted participants (Fig. 4). Our source analyses estimated this effect to be generated primarily within right superior frontal lobe, overlapping with right premotor cortex. A subsequent memory contrast within each cluster indicated significantly larger ERPs for subsequent hits relative to subsequent misses in the attentive group, with the Bayes factor in the frontal cluster indicating substantial evidence for a subsequent memory effect [positive frontal cluster: *t*(12) = 2.657, *P* = 0.021, BF10 = 3.195; negative posterior cluster: *t*(12) = −2.320, *P* = 0.039, BF10 = 1.963].

**Figure 5.**
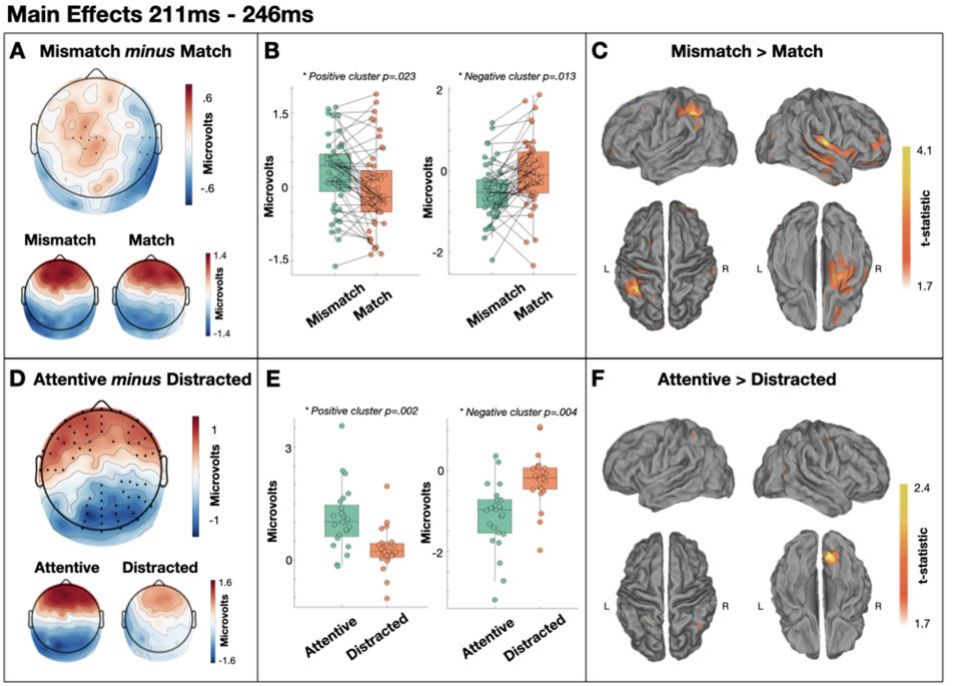
Main effects of target type and attention from 211 to 246 ms. (**A**) Scalp distribution of the significant effect of target type. Electrodes contributing to the clusters are marked. (**B**) Single-subject mean voltages within each significant cluster. (**C**) Estimated sources of the main effect within left supramarginal gyrus and right insula. (**D**) Scalp distribution of the significant effect of attention. Electrodes contributing to the clusters are marked. (**E**) Single-subject mean voltages within each significant cluster. (**F**) Estimated sources of the main effect within right visual cortex

We also observed a dipolar main effect of target type in the 211–246 ms time window with a larger left frontocentral positivity to mismatched targets than to matched targets (cluster *P* = 0.023) and a larger right temporal negativity to mismatched targets than to matched targets (cluster *P* = 0.013; Fig. 5A–C). Source analyses estimated this effect to be primarily generated within left supramarginal gyrus and right insula. Both Frequentist and Bayesian *t*-tests indicated no compelling evidence of subsequent memory effects in the attentive group in either cluster [positive cluster: *t*(12) = −1.764, *P* = 0.103, BF10 = 0.939; negative cluster: *t*(12) = 1.964, *P* = 0.073, BF10 = 1.210].

In the same time window (211–246 ms), we also observed a main effect of attention, with greater frontal positivity (cluster *P* = 0.002) and greater posterior negativity (cluster *P* = 0.004) in the attentive group relative to the distracted group, with estimated generator in right visual cortex (Fig. 5D–F). As with the effect of target in this time window, both Frequentist and Bayesian *t*-tests agreed that there is no evidence of subsequent memory effects in either cluster [positive cluster: *t*(12) = 1.557, *P* = 0.145, BF10 = 0.734; negative cluster: *t*(12) = −1.443, *P* = 0.175, BF10 = 0.647].

**Figure 6.**
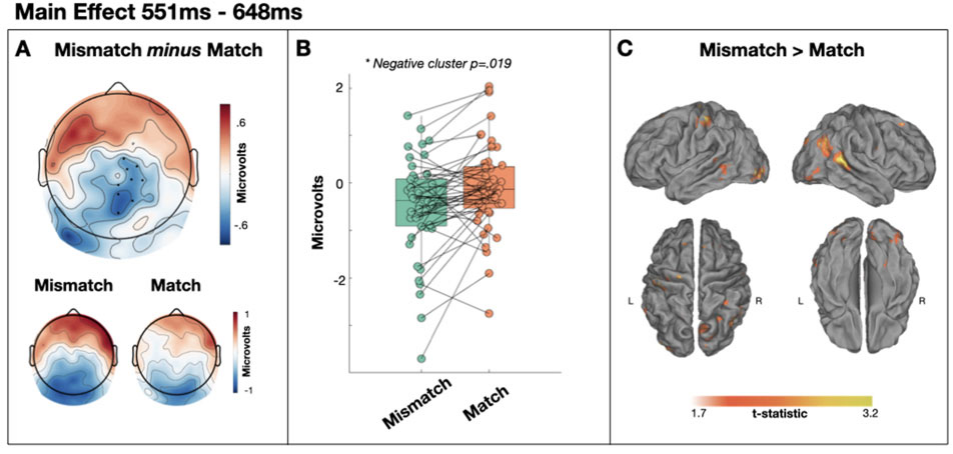
Main effect of target type from 551 to 648 ms. (**A**) Scalp distribution of the significant effect. Electrodes contributing to the cluster are marked. (**B**) Single-subject mean voltages within the significant cluster. (**C**) Estimated sources of the main effect within right posterior superior temporal gyrus

In the 551–648 ms time window, we observed an effect of target type, with a larger centroparietal negativity to mismatched targets than matched targets (cluster *P* = 0.019) estimated to be generated in the right posterior superior temporal gyrus (Fig. 6). The subsequent memory contrast in this cluster failed to reach our significance threshold, and the Bayesian equivalent similarly concluded only anecdotal evidence in favour of the hypothesis [*t*(12) = 2.158, *P* = 0.052, BF10 = 1.568].

The main effect of target in the 137–207 and 652–707 ms time windows did not pass our significance threshold (*P* = 0.048 and 0.042, respectively; alpha = 0.025). The main effect of attention in the 375–547 ms, and 652–707 ms time windows also did not pass our significance threshold (*P* = 0.047 and 0.082, respectively; alpha = 0.025). No clusters were formed in any other contrast or time window.

## Discussion

Consistent with our hypothesis and previous research, repetition priming enhanced the perceptual intelligibility of the degraded targets (Davis *et al.* 2005; Hervais-Adelman *et al.* 2012; Wild *et al.* 2012b; Sohoglu *et al*. 2014). This result is, independently, evidence for the importance of prior knowledge (or expectation) for generating a conscious experience of comprehending degraded speech—i.e. a ‘pop-out’. Furthermore, consistent with a proposed two-stage ERP profile of auditory processing (Rohaut *et al.* 2015), we observe two dissociable ERP effects.

**Figure 7.**
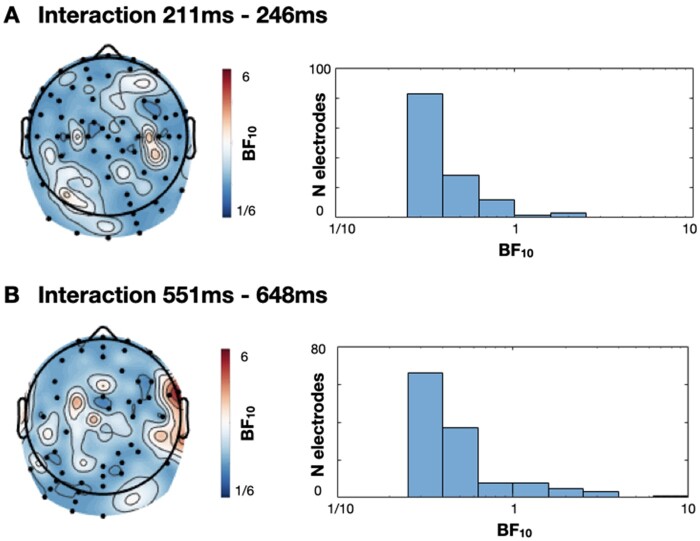
Scalp distribution of Bayes Factors (from Bayesian equivalent *t*-tests) in tests of the interaction between target type and attention in time windows (**A**) 211 to 246 ms and (**B**) 551 to 648 ms. All electrodes with Bayes Factors >3 or <⅓ are marked on the scalp. Histogram shows the distribution of Bayes Factors across the head

First, and contrary to some arguments of attentional enhancement of prediction errors (Auksztulewicz and Friston 2015), we observe an early predictive signal (211–246 ms)—i.e. larger for unpredicted words than for predicted words—that does not significantly interact with attention. Indeed, the results of our Bayesian analysis of this effect indicate considerable evidence for the absence of interaction with attention (see Fig. 7A). Specifically, 96% of electrodes provided greater evidence for the null hypothesis of no interaction between target type and attention in this time window (i.e. BF < 1), 48% of which provided substantial evidence for the null (i.e. BF < ⅓). Within a two-stage model of auditory processing, this effect may be analogous to the mismatch negativity, which has a similar time course and can be elicited by rare stimuli without attention or conscious awareness (Heilbron and Chait 2018). However, we had not expected to find evidence for differential processing of matched and mismatched targets during inattention—a result that is seemingly inconsistent with prior evidence that successful comprehension of degraded speech requires top-down expectations (e.g. Wild *et al.* 2012b). Indeed, inattentive participants should have been unable to form top-down expectations that would subsequently elicit a prediction error. One parsimonious interpretation is that the distraction task did not sufficiently direct attention away from the speech stimuli, thus allowing those participants the opportunity to also generate expectations while completing the visual distraction task. However, a Bayesian analysis indicated that our data provide substantial evidence that distracted participants’ memory for the mismatched prime words did not differ from zero. As all prime words were heard as clear speech, we would expect that memory would be above chance here if the participants were not sufficiently distracted. Therefore, we conclude that the early differential processing of targets during inattention is not the result of insufficient inattention.

An alternative interpretation is that the signal reflects the error of a non-conscious expectation that can be generated without top-down influence. For example, previous studies of word-pair priming of noise-vocoded speech have used the written form of the word as the prime stimulus, whereas our prime stimuli were clear (non-degraded) versions of the same speech stimulus. Therefore, it is possible that a low-level prediction that an auditory stimulus will have the same envelope as the just-heard auditory stimulus, e.g. could be generated inattentively. Indeed, prediction error minimization accounts posit that expectations are generated at multiple levels of the processing hierarchy. Consequently, while a conscious top-down expectation may not be generated by an inattentive participant, an expectation from a lower level of the hierarchy may nevertheless be instantiated and compared with the sensory input—e.g. an expectation that the auditory environment will remain stable, as is one interpretation of the mismatch negativity during inattention (Sussman and Winkler 2001). Indeed, our source analyses estimate generators of this effect primarily within right temporal lobe and left supramarginal gyrus (see Fig. 5C), while a similar previous study involving visual primes (rather than auditory primes as in this study) reported an effect with a similar time course to be generated within the more canonically semantic regions of left middle and inferior frontal gyri (Sohoglu *et al.* 2012). We therefore suggest that this effect is a non-semantic error signal, while similar studies that involve visual primes and auditory targets may be more likely to promote semantic expectations. Indeed, the estimated right temporal lobe generator of our error effect is also linked to domain-general processing of complex auditory stimuli, rather than to specific linguistic and semantic processing (McGettigan and Scott 2012).

As hypothesized, we also observe a later component that is largest for degraded words that ‘pop-out’ into awareness. Specifically, from ∼250 to 350 ms post-stimulus the ERPs are larger for matched targets than mismatched targets in the attentive group only. Indeed, in that same time window, the ERPs in the distracted group exhibited a similar distribution to the preceding error signal (Fig. 3D). The apredictive nature of this ERP component is seemingly at odds with prediction error accounts of evoked potentials. However, the concept of precision is often used to explain such patterns (Kok *et al.* 2012). Specifically, the error signal is considered to be weighted by the system’s confidence in that signal—its precision. Attention is one mechanism that is thought to increase precision (Hohwy 2012). Therefore, one could argue that while a fulfilled prediction about an upcoming word elicits little prediction error, an individual’s attention to the word increases precision which multiplicatively leads to a larger precision-weighted prediction error signal (i.e. an evoked potential) than an unpredicted but unattended stimulus. Indeed, the effect of attention to boost the magnitude of evoked potentials is evident in the two main effects of attention we observe prior to this effect (137–207 and 211–246 ms; see Figs 4 and 5). However, it is clear that any observed form of interaction (i.e. predictive or apredictive) can be explained by appealing to the multiplication of two hidden and independently varying signals (namely, error and precision), thus creating issues in rigorously studying the role of precision weighting in perception (cf. Heilbron and Chait 2018). Nevertheless, under a precision-weighted prediction error interpretation, some argue that all evoked potentials should interact with attention (e.g. Heilbron and Chait 2018), which is demonstrably not the case for our earlier main effect of target (211–246 ms; see Fig. 7) unless one appeals to complex *post hoc* interactions of precision and error. Nevertheless, a pivotal challenge for the influence of precision weighting on prediction error signals would come from evidence of entirely independent influences of prediction and attention on evoked potential amplitudes.

Under a Global Neuronal Workspace interpretation, this later component could be considered to reflect the breakthrough of a stimulus representation into conscious experience (Alsufyani *et al.* 2019). While we did observe weak evidence of recollection of mismatched targets (*P* = 0.020; BF10 = 2.720), indicating that mismatched targets were not entirely unintelligible perhaps due to a degree of perceptual learning across the experiment (Hervais-Adelman *et al.* 2008), attentive participants’ ratings of intelligibility (noisiness) were entirely consistent with the pop-out of meaning following matched primes (Davis *et al.* 2005). Furthermore, this component was larger for subsequently remembered items than for subsequently forgotten items, albeit weakly (*P* = 0.049; BF10 = 1.628). The link to subsequent successful recognition provides further evidence to link this ERP component with a conscious experience on the part of the listener. However, this effect is earlier than we predicted based on a typical two-stage profile and its scalp distribution is more reminiscent of a P3a than the P3b or other late positive components typically linked to global-workspace breakthrough effects. Nevertheless, P3a-like components have been observed in breakthrough contexts (Bowman *et al.* 2013) and Global Neuronal Workspace theory broadly posits that scalp positivities, as observed here, reflect the ignition of a representation into conscious access (Dehaene and Christen 2011). Source estimates of other late positivities within this framework typically involve generators distributed across the cortex, consistent with a brain-wide ignition into conscious access (e.g. Bekinschtein *et al.* 2009). However, while the source estimate of our observed pop-out effect includes some weak evidence of generators distributed across lobes and hemispheres (see Fig. 3C), the focus is estimated to be in the right middle temporal gyrus and right fusiform gyrus. Interestingly, the late positivity described by Rohaut *et al.* (2015), and linked to the stage of conscious access of meaning within the two-stage profile, was also estimated to be generated within right fusiform gyrus, as well as left dorsolateral frontal cortex. Furthermore, there is evidence for greater activity within the right fusiform gyrus when the meaning of speech is task-relevant (von Kriegstein *et al.* 2003). We conclude therefore that our ERP positivity reflects conscious access of the meaning of speech. While we cannot rule out the potential role of task-related post-perceptual processing rather than conscious access itself (Aru *et al.* 2012), we argue that the majority of evidence for components linked to such processes occur later in time than the pop-out effect observed here (i.e. after ∼350ms; e.g. Pitts *et al.* 2014; Schelonka *et al.* 2017).

In a later time window, from ∼550 to 650 ms, we also observed more extreme ERPs for mismatched targets than for matched targets that did not interact with attention (see Figs 6 and 7). At first glance, it is unclear why post-breakthrough processing should differ according to the relative match of expectations. The scalp distribution of this effect is markedly similar to that reported in an overlapping time window of 450 to 700 ms post-target by Sohoglu *et al.* (2012; see Fig. 4B in that paper) who also found that magnetoencephalography (MEG) sensor data in the same time window significantly predicted trial-by-trial ratings of speech clarity, such that reduced neural responses to matched targets were accompanied by increased experiences of speech clarity. Our source estimates indicated a primarily right posterior superior temporal generator for this effect, while Sohoglu *et al.* (2012), with more sensitive MEG source analyses, report right temporal generators alongside bilateral inferior frontal and middle occipital gyri. Sohoglu *et al.* (2012), therefore, conclude that this effect reflects the neural processes that generate the experience of speech clarity. On that basis, we would expect this effect to interact with attention in this study. However, we find no evidence for this interaction. Nevertheless, while Bayes equivalent *t*-tests at each electrode in this time window indicated substantial evidence for no interaction in the majority of electrodes, two electrodes did exhibit substantial evidence for an interaction (i.e. BF10 > 3; Fig. 7B). As our cluster forming threshold required four neighbouring electrodes, it is possible that this effect does indeed interact with attention, but to an extent that is not evident with our specific analysis choices. If that were the case then, these later effects may indeed reflect processes associated with the conscious experience of meaning, or may reflect consequent processes such as those in service of task demands—i.e. providing a judgement of the noisiness of the stimulus (Aru *et al.* 2012). Indeed, one might expect that, if evoked potentials reflect breakthrough into awareness, as we have argued, subsequent post-breakthrough potentials would reflect cognitive operations upon that percept, such as response selection or meta-cognition. This would favour a three-stage account of evoked potentials—(i) an early prediction error processing stage, (ii) a subsequent breakthrough into awareness and (iii) a consequent stage of cognitive operations in support of task goals. These stages are reminiscent of characterizations of neural correlates of consciousness into the prerequisites and consequences of conscious perception, as well as the perceptual experience itself (Aru *et al.* 2012). However, given our lack of strong evidence either for or against an interaction with attention, the functional significance of our 551–648 ms effect remains a target for future investigation.

One potential formulation of the way in which we might expect prior knowledge and attention to interact is for greater precision to lead to accelerated processing, and therefore earlier ERP latencies. Variable latencies across conditions may thus create spurious amplitude differences due to temporal smearing. Following the suggestion of an anonymous reviewer, we tested the interactive effects of expectation and attention on the latencies of each of our identified components. Crucially, our analyses provided evidence in favour of the hypothesis that component latencies did not interact between expectation and attention (all BFinclusion between 0.252 and 0.523; see Supplementary data), thus indicating that our observed amplitude differences are unlikely to be driven by differential effects of precision/expectation latency. Nevertheless, more advanced multivariate analyses across multiple levels of speech degradation may further delineate the ERP correlates of precision-weighted prediction error from those of putative breakthrough effects (see Blank and Davis 2016).

## Conclusions

Our results indicate a link between the conscious experience of semantic pop-out in comprehension of degraded speech and a positive-going ERP in the range of 300 ms post-stimulus—consistent with a Global Neuronal Workspace framework. Prior to this positivity, ERPs appear to reflect the error of non-semantic predictions, consistent with prediction error minimization accounts. To consider our observed late positivity within the same prediction error account requires a *post hoc* appeal to freely varying precision weighting that is not straightforwardly verified. We therefore suggest that our data are consistent with early negative-going ERPs as reflections of prediction error while later positive-going ERPs reflect conscious access and processes in support of task demands (e.g. Dehaene and Christen 2011; Rohaut *et al.* 2015).

## Supplementary data

Supplementary data is available at *NCONSC Journal* online.

## Supplementary Material

niaa022_Supplementary_Data
