# Supplementary material for: Event-related potentials reflect prediction errors and pop-out during comprehension of degraded speech
Source: Neurosci Conscious. 2020 Oct 25;2020(1):niaa022. doi: 10.1093/nc/niaa022 (PMC7585676; doi:10.1093/nc/niaa022)
Supplement: niaa022_Supplementary_Data [file niaa022_supplementary_data.zip › Supplementary Table 3.docx]

*Supplementary Table 3. Summary of recognition memory results: Two-way mixed ANOVAs and equivalent Bayesian ANOVAs, with factors of word type (clear prime; degraded target) and attention (attentive; distracted).*

|  | F | *p* | BFinclusion |
| --- | --- | --- | --- |
| **Discrimination (d’)** |  |  |  |
| Word type | 30.243 | <.001 | 8706.165 |
| Attention | 8.714 | 0.005 | 8.053 |
| Interaction | 0.528 | 0.471 | 0.358 |
| **Recollection** |  |  |  |
| Word type | 13.287 | <.001 | 60.743 |
| Attention | 2.411 | 0.127 | 1.735 |
| Interaction | 0.129 | 0.721 | 0.160 |
| **Familiarity** |  |  |  |
| Word type | 14.533 | <.001 | 42.913 |
| Attention | 1.569 | 0.217 | 0.666 |
| Interaction | 0.496 | 0.485 | 0.323 |
