# Supplementary material for: Event-related potentials reflect prediction errors and pop-out during comprehension of degraded speech
Source: Neurosci Conscious. 2020 Oct 25;2020(1):niaa022. doi: 10.1093/nc/niaa022 (PMC7585676; doi:10.1093/nc/niaa022)
Supplement: niaa022_Supplementary_Data [file niaa022_supplementary_data.zip › Supplementary Table 1.docx]

*Supplementary Table 1: Mean and standard deviation (in brackets) of the word list characteristics: frequency (ln[BNC]), imageability, length in phonemes, and length in letters.*

|  | Frequency | Imageability | Length (ph) | Length (L) |
| --- | --- | --- | --- | --- |
| Word List 1 | 1.92 (1.29) | 566.6 (55.59) | 3.29 (0.54) | 4.14 (0.76) |
| Word List 2 | 1.97 (1.31) | 577.9 (53.3) | 3.33 (0.58) | 4.14 (0.76) |
| Word List 3 | 2 (1.3) | 570.3 (47.53) | 3.28 (0.48) | 4.14 (0.76) |
| Word List 4 | 2.1 (1.28) | 563.8 (56.24) | 3.33 (0.47) | 4.14 (0.76) |
