# Supplementary material for: Event-related potentials reflect prediction errors and pop-out during comprehension of degraded speech
Source: Neurosci Conscious. 2020 Oct 25;2020(1):niaa022. doi: 10.1093/nc/niaa022 (PMC7585676; doi:10.1093/nc/niaa022)
Supplement: niaa022_Supplementary_Data [file niaa022_supplementary_data.zip › Supplementary Table 2.docx]

*Supplementary Table 2. ANOVAs and equivalent Bayesian ANOVAS testing for differences across lists at each word characteristic.*

|  | F | *p* | BF10 |
| --- | --- | --- | --- |
| Frequency | 0.233 | 0.873 | 0.021 |
| Imageability | 0.779 | 0.507 | 0.054 |
| Length (ph) | 0.217 | 0.885 | 0.021 |
| Length (L) | <.001 | 1 | 0.016 |
