# Supplementary figures and images for: Event-related potentials reflect prediction errors and pop-out during comprehension of degraded speech
Source: Neurosci Conscious. 2020 Oct 25;2020(1):niaa022. doi: 10.1093/nc/niaa022 (PMC7585676; doi:10.1093/nc/niaa022)

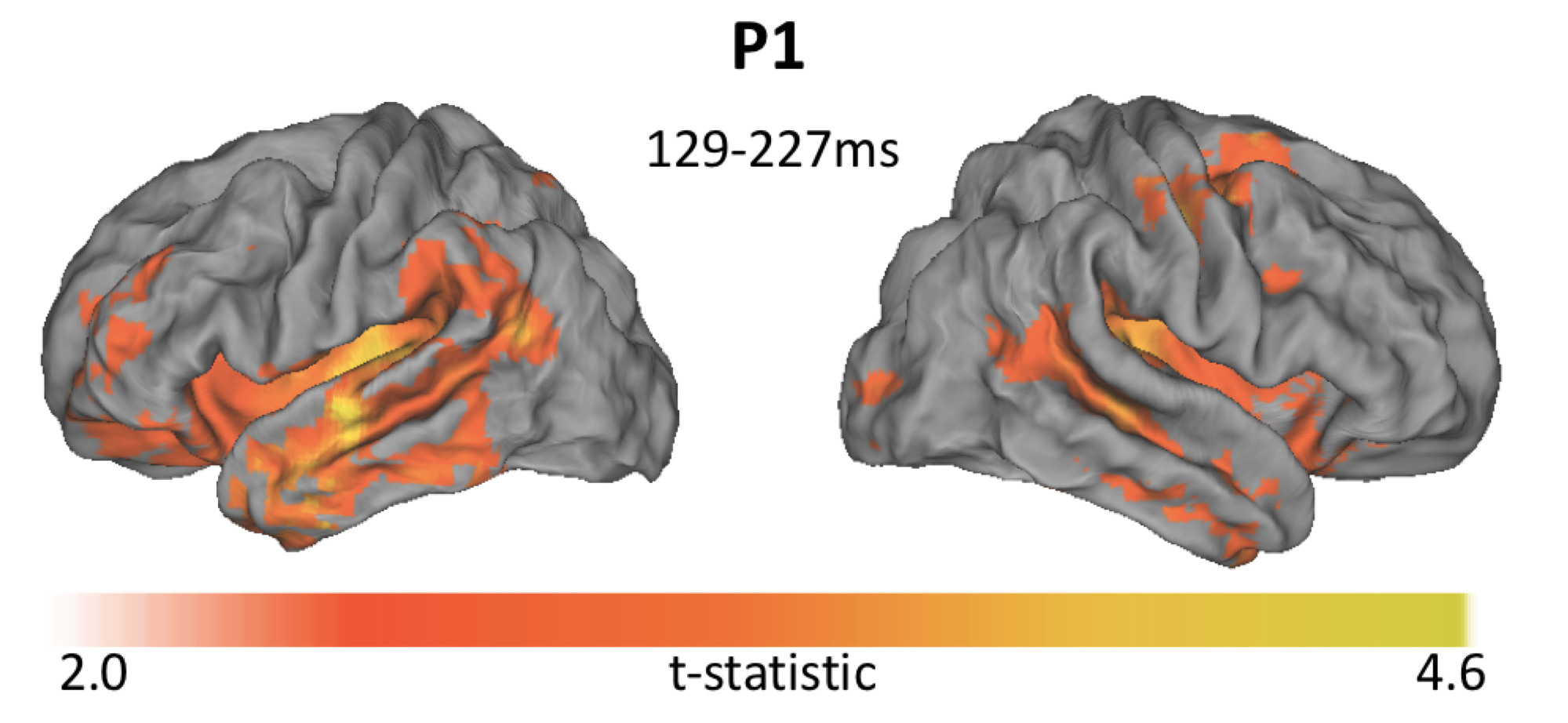

Supplement: niaa022_Supplementary_Data [file niaa022_supplementary_data.zip › SupFig1.jpg]
